# Validation and extraction of molecular-geometry information from small-molecule databases

**DOI:** 10.1107/S2059798317000079

**Published:** 2017-02-01

**Authors:** Fei Long, Robert A. Nicholls, Paul Emsley, Saulius Gražulis, Andrius Merkys, Antanas Vaitkus, Garib N. Murshudov

**Affiliations:** a Structural Studies, MRC Laboratory of Molecular Biology, Francis Crick Avenue, Cambridge CB2 0QH, England; b Institute of Biotechnology, Saulėtekio al. 7, LT-10257 Vilnius, Lithuania

**Keywords:** validation, high-order statistics, Crystallography Open Database

## Abstract

A freely available small-molecule structure database, the Crystallography Open Database (COD), is used for the extraction of molecular-geometry information on small-molecule compounds. The results are used for the generation of new ligand descriptions, which are subsequently used by macromolecular model-building and structure-refinement software. To increase the reliability of the derived data, and therefore the new ligand descriptions, the entries from this database were subjected to very strict validation. The selection criteria made sure that the crystal structures used to derive atom types, bond and angle classes are of sufficiently high quality. Any suspicious entries at a crystal or molecular level were removed from further consideration. The selection criteria included (i) the resolution of the data used for refinement (entries solved at 0.84 Å resolution or higher) and (ii) the structure-solution method (structures must be from a single-crystal experiment and all atoms of generated molecules must have full occupancies), as well as basic sanity checks such as (iii) consistency between the valences and the number of connections between atoms, (iv) acceptable bond-length deviations from the expected values and (v) detection of atomic collisions. The derived atom types and bond classes were then validated using high-order moment-based statistical techniques. The results of the statistical analyses were fed back to fine-tune the atom typing. The developed procedure was repeated four times, resulting in fine-grained atom typing, bond and angle classes. The procedure will be repeated in the future as and when new entries are deposited in the COD. The whole procedure can also be applied to any source of small-molecule structures, including the Cambridge Structural Database and the ZINC database.

## Abstract 2

The entries from a freely available small-molecule database, the Crystallography Open Database, have been validated and a reliable subset of molecules has been selected for the extraction of molecular-geometry information. The atom types and corresponding bond and angle classes derived from this database have been subjected to validation, the results of which are used by *AceDRG* in the derivation of new ligand descriptions.

## 1. Introduction   

Small-molecule databases such as the Cambridge Structural Database (CSD; Groom *et al.*, 2016 ▸) and the Crystallography Open Database (COD; Gražulis *et al.*, 2012 ▸) are a rich source of information that can be used for various purposes, including the extraction of molecular-geometry information and its use for the generation of new ligand descriptions (Engh & Huber, 1991 ▸; Parkinson *et al.*, 1996 ▸; Bruno *et al.*, 2004 ▸; Long *et al.*, 2017 ▸; Moriarty *et al.*, 2009 ▸; Emsley *et al.*, 2010 ▸). However, the entries in these databases have been generated by experimental techniques and the coordinates are models describing these experiments. The influence of human factors affecting the reliability of derived atomic models should not be ignored. As is the case for any models derived from experimental observations, models are prone to errors and include mis­interpretations. There might be a multitude of reasons for errors/misinterpretations of the entries in these databases, including deficiencies in the experimental data (*e.g.* systematic and random errors during data acquisition), the software used, mismodelling of the experiment and, finally, plain cheating. The entries from these databases must be validated using criteria that are as strict as possible and selected with extreme care, as the derived data are likely to be used by many structural biologists for the refinement of macromolecular structures. The resulting macromolecular coordinates are deposited in the PDB (Berman *et al.*, 2002 ▸) and are further used by the wider community of biologists. In many cases, the coordinates from the PDB are considered to be accurate and serve as observations for further classifications and analyses. If extreme care is not exercised and inaccurate molecular-geometry information is used in deriving coordinates then the errors can persist, and may affect future results and conclusions. Subsequently validation and cleaning up of such errors might become even more challenging than it is now. This puts an additional responsibility on software developers and, in particular, designers of molecular-geometry databases.

There are already a number of software tools for small-molecule validation. Validation starts during structure solution and refinement (Sheldrick, 2008 ▸). The small-molecule structures must be validated using tools such as *checkCIF* (Spek, 2009 ▸) before they are deposited in a database. The structures in the CSD are regularly checked and updated by various authors (Herbstein & Marsh, 1998 ▸; Marsh & Spek, 2001 ▸) and by the CCDC staff (Groom *et al.*, 2016 ▸). Rarely does this happen for the COD. Usually, data deposited in the COD are subjected to less validation than those deposited in the CSD. One reason for this is that the COD is relatively young and is still gathering pace. Nevertheless, the sheer number of entries in the COD (more than 366 000 as of September 2016) makes it possible to derive sufficiently accurate molecular-geometry information. Strict validation of the COD entries ensures that the derived data are of sufficiently high quality and can be used by the structural biology community with confidence. It should be noted that additional validation must be applied to any database before deriving data for such purposes; we would not like to add erroneous information to the PDB, as the PDB has enough problems of its own as it is (Joosten *et al.*, 2012 ▸; Pozharski *et al.*, 2013 ▸; Weichenberger *et al.*, 2013 ▸; Reynolds, 2014 ▸; Malinska *et al.*, 2016 ▸).

The main aim of this contribution is to describe the validation tools used for selection of entries from the COD, and the validation of bond lengths and angles. The selected entries are used to derive local chemistry-based and topology-based atom types that are used to generate bond and angle classes. These tables, together with atom-type determination, are used by *AceDRG* (Long *et al.*, 2017 ▸) to generate molecular-geometry information for new ligands. The generated data, at its various levels, can be made available for software developers for further usage. Atom, bond and angle class tables are already available from *CCP*4 under the LGPL license.

*Organization of this paper*. Firstly, we briefly describe the selection and validation of COD crystal structures and derived molecules, and how the derived atom types, bonds and angles are subjected to further validation. We then move on to a description of each step in detail, before describing the feedback cycle from statistical validation to fine-tune atom types, bond and angle classes. At the end, we conclude with a summary of the current state and our views on future developments.

## 2. Overview of validation and entry selection from the COD   

Entries from the COD and derived data are subjected to four main stages of validation before they are accepted for further use. These are as follows.

(i) Validation of the database of small molecules. This is performed when the data are deposited in the COD. Gražulis *et al.* (2009 ▸) have described this step in detail, and thus we mention this stage here only briefly.
(ii) *AceDRG* validation of coordinate CIF files and generated molecules.
(iii) *AceDRG* validation of derived atom types, bonds and angles.
(iv) Statistical validation of the data produced by *AceDRG*. This step is performed iteratively together with revisions of the atom-typing protocol. The results of validation are fed back to *AceDRG* to help with the fine-tuning of atom types.

## 3. Validation   

As described by Long *et al.* (2017 ▸), the atom-type classes extracted by *AceDRG* from a small-molecule database (*e.g.* the COD) are used to classify bonds and angles. These classes are used to generate molecular-geometry information for new ligands. The validity of the tables, and thus the molecular-geometry information generated for a particular ligand, depends on the reliability of the crystal structures used to originally derive these tables, as well as on the suitability of the atom types. Although the number of atom-type classes derived by *AceDRG*, which encapsulate the local chemical environments of atoms, is large (more than 260 000, see Table 1 ▸), not all of chemical structure space will be covered.

### 3.1. Validation of deposited data   

There are a number of criteria used to validate structures deposited in the COD. These can be found in Gražulis *et al.* (2009 ▸) and on the COD website (http://www.crystallography.net/cod/). Briefly, all structure CIFs that are deposited in the COD are first of all subjected to an automated check of syntactic correctness. This step, although purely technical and automatic, is very important as it enables all further automated processing and ensures the unambiguous extraction of CIF data. Syntactically deficient files are either corrected (most corrections are automatic, although manual corrections are performed on a best-effort basis), or they are rejected from deposition. Further, files are checked for the presence and correctness of essential data (for example, a check is performed to make sure that all three *x*, *y* and *z* coordinates of atoms are present). For personal communications and pre-publication structures, quality of the structure-convergence parameters is checked against the criteria specified by the IUCr (ftp://ftp.iucr.org/pub/dvntests); structures are rejected if they are in a ‘red’ zone of the parameter deviations. For published structures such checks are recorded but not enforced, assuming that a published structure had a good reason to bypass quality indicators.

### 3.2. Selection of crystal data   

To ensure high quality of the derived molecular-geometry data, *AceDRG* selects crystals using the following criteria.

(i) *Experimental method*. The selected coordinates must correspond to crystallographic diffraction data. Unfortunately, the COD entries do not explicitly define the method that was used to derive coordinates. Therefore, the following approach is used. (1) If there is a publication detail that contains ‘powder diffraction’, or if there is an item containing _pd_, then the structure entry is excluded from further consideration. We also exclude entries that do not contain _diffrn_reflns_ anywhere in the CIF file. As a result of these filters, we excluded 4184 entries.
(ii) *Resolution of the data*. The small-molecule CIF entries from the COD do not seem to explicitly contain information about the high-resolution limit. We calculate the highest resolution using information provided by the items _diffrn_radiation_wavelength and _diffrn_reflns_theta_max. Specifically, the maximum resolution is calculated using Bragg’s formula: *d*_max_ = λ/(2sinθ_max_). If *d*_max_ > 0.84 or these items are not defined then the entry is excluded from further consideration. This is similar to the criterion recommended for publication in IUCr Journals and to that used by *checkCIF* (Spek, 2009 ▸) in the validation of small-molecule CIF files.
(iii) *Quality*. There may be many different incarnations of annotations of *R* factors in any given COD entry. Therefore, any occurrences of the mention of an *R* factor, such as _refine_ls_R_factor_all and _refine_ls_R_factor_gt, are searched and the corresponding values are extracted. If any of these values are greater than 0.1, or there are no references to *R* factors, then the crystal structure is excluded from the list. The number of structures excluded using the *R*-factor criterion was 134 322.

The number of remaining crystal structures after filtering the COD entries using the above criteria was 167 489 (see Table 2 ▸ *a* for details).

### 3.3. Selection of molecules   

The crystal structures that pass the initial selection criteria are then used to generate molecules. All symmetry operators are used to generate connected graphs that are assumed to be the molecule(s). Currently, for molecule generation 3 × 3 × 3 unit cells of a crystal are used. If a molecule has infinite length (such as graphene) then the molecule is truncated.

The purpose of this validation stage is to remove disordered and wrong molecules, as their inclusion can distort bond-length and angle distributions.

**Figure 1:**
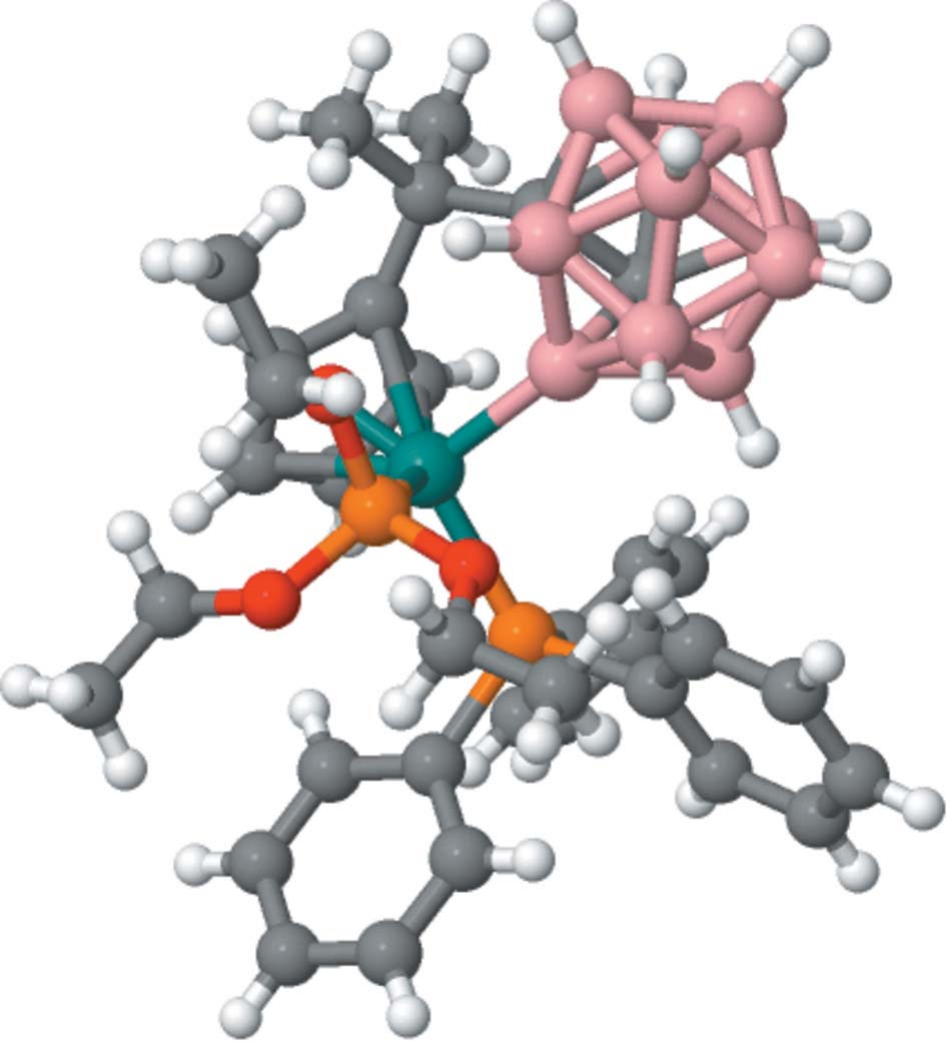
An example of carbon with more than four connections. The COD code for this structure is 4108751. It is a perfectly valid structure that will be dealt with in due course. This figure was produced by *Jmol* (http://www.jmol.org/).

**Figure 2:**
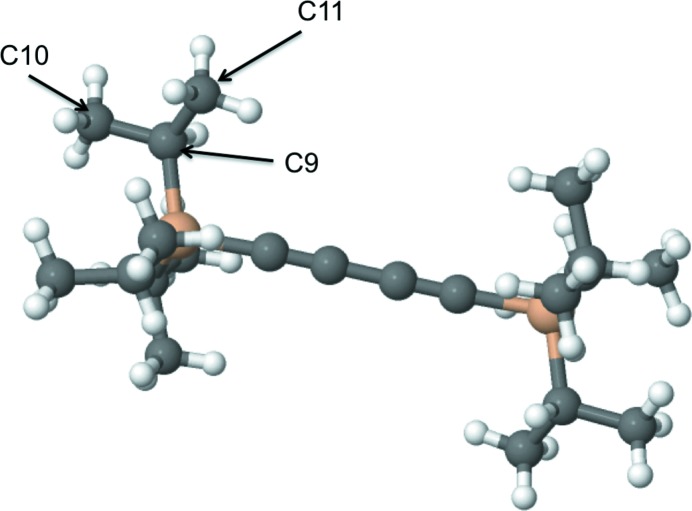
An example where exactly the same atom-type pairs have different bond lengths: COD code 2015492. The distance between C9 and C10 is 1.52 Å, whereas the distance between C9 and C11 is 1.43 Å. Both C10 and C11 have exactly same chemical environment and topology, and therefore the same atom type. This figure was produced by *Marvin Sketch* (http://www.chemaxon.com).

(i) During molecule generation, if one of the symmetry-generated atoms collides with any other atom, *i.e.* the distance between them is less than 0.1 Å, then the whole crystal is excluded from further consideration.
(ii) At this stage, connected graphs are identified using an equivalent class identification algorithm (Press *et al.*, 1992 ▸), and each connected component is considered as a molecule. If the distance *r* between two atoms in a crystal is within the range *r*_min_ < *r* < *r*_max_ then these atoms are considered as connected. For each atom, there is a valence radius (Table 3 ▸). These radii are used to determine whether an atom pair is connected *via* a bond. Distance ranges are calculated using the formulae
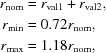
where *r*_val1_ and *r*_val2_ are the valence radii of the two atoms under consideration, *r*_nom_ is the nominal bond length, which is roughly equal to the single-bond distance between the two atoms, and *r*_min_ and *r*_max_ specify the range used to verify the existence of bonds between atoms.If there is at least one interatomic distance that is less than *r*_min_ then the whole molecule is excluded from further consideration.If a molecule thus generated has one or more atoms with occupancy less than 1 then this molecule is excluded from the list. Note that the asymmetric unit of a crystal may contain more than one molecule; it may happen that only some of the generated molecules from a given crystal are rejected. See Table 2 ▸(*b*) for details.
(iii) For each atom, the number of connections to the atom is checked for consistency with its valence. If there are any inconsistencies then the molecule is excluded from the list. Table 4 ▸ gives details of the maximum allowed valences for atoms in the extended organic set. Fig. 1 ▸ shows one example of a molecule excluded according to this criterion: it is a perfectly valid carborane-type molecule, although the current version of *AceDRG* does not deal with this type of molecule, and thus related molecules are excluded from further calculations.After molecules have been generated and selected for further use, the atom types described by Long *et al.* (2017 ▸) are generated for each atom for each molecule. All bonds and angles are also generated. During molecule generation, we keep information about symmetry. Bond lengths and angles are added to the list if at least one of the atoms involved are within the asymmetric unit.
(iv) If the bond length for a particular atom-type pair within a molecule varies by more than 0.02 Å then we exclude the whole molecule (Fig. 2 ▸). If the bond lengths for different or same combinations of atom types within a given molecule are the same then the whole molecule is excluded. The purpose of this criterion is to ensure that the results of (wrongly) constrained refinements are not used. COD entry 1100779 is an example of where bonds between different atom-type pairs containing different elements may have been constrained to be the same.
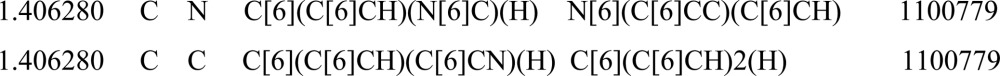
An example where similar atom-type pairs have the same bond lengths is COD entry 7203601.
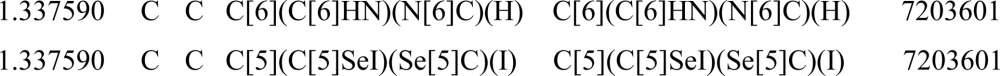
The remaining molecules are then used to generate the raw bond and angle lists. These bonds and angles are subjected to further validation as described below. Table 2 ▸(*b*) gives details of the selection of the molecules.

### 3.4. Independent validation of the derived tables   

Once atom types and the bond and angle tables have been generated, they are validated using statistical tools. The purpose of this validation step is twofold: firstly to clean up the tables and secondly to feed back unusual behaviour to help redefine atom types and further fine-tune atom-type classes. It should be noted that *AceDRG* produces two tables for bonds (and likewise for angles).

(i) The raw list of all bonds, containing references to the crystal structures that they came from. This table is used internally for validation purposes. This is essentially the list of observed bond lengths with reference to the atom-type classes (Long *et al.*, 2017 ▸). We call this table the ‘bond observation table’.
(ii) The reduced and reorganized bond tables that are used by *AceDRG* to generate molecular-geometry information on the fly for ligands of interest. This table is distributed by *CCP*4, and is used by *AceDRG* when invoked in ligand description-generation mode. We call this table the ‘bond class table’.

Since in this work we do not consider H atoms as observables, and do not trust their bond lengths (it is highly likely that H atoms have been added in the riding positions for most of the entries generated by small-molecule crystallography; Sheldrick, 2008 ▸), we ignore them in the following analyses. Although accurate positions of protons can be derived using high-resolution neutron diffraction experiments (Allen & Bruno, 2010 ▸), in this work we do not consider them. The bond-length distribution corresponding to the protons and electrons of H atoms will be a subject of future work.

At this stage of validation, we perform the following checks.

(i) Consistency of bond class and bond observation tables. This step is essentially for debugging *AceDRG*-generated tables, and therefore the algorithms implemented in the program.
(ii) Within the bond class table we check for uniqueness of atom classes at various levels of hierarchy.
(iii) Within the bond observation table we identify unclassified bonds. It is likely that the number of bonds corresponding to some of the atom-type pairs is too small (less than four) to be included in the final bond class table.

### 3.5. Statistical analysis of bond tables   

For statistical analysis, the following properties are used.

(i) *The standard deviation of bond lengths in the bond class table.* Bond classes with particularly small standard deviations were identified and flagged for further analysis. It is likely that very small standard deviations are the result of bond-constrained refinement, and using bond lengths and standard deviations for such classes would result in biased molecular-geometry values. If the standard deviation for a particular bond class is too large then it is likely that the atom types need to be further refined or that the structures that they were calculated from are wrong. The extreme cases were checked manually; either the molecules were removed from further consideration, validation criteria were added to account for such behaviour or the atom types were refined further in light of this information.
(ii) **Z*-scores.* If a particular observed value deviates from the mean by more than 5σ then it is likely that either this value came from the wrong structure or that it must be represented by another bond class. Entries and atom types corresponding to the few highest *Z*-scores were inspected manually; either the offending molecule was removed from further consideration or the atom types were updated to account for these differences.
(iii) *Higher-order moments.* We used skewness (based on the third moment) and kurtosis (based on the fourth moment) to identify unusual distributions of distances. Whilst the distribution of distances cannot be considered to be Gaussian in general, it is a good enough approximation in the vicinity of the maximum of the distribution. Since the atom and bond classes we are interested in are in the vicinity of the maximum of the distribution, distributions that are particularly skewed or heavy-tailed are considered to be suspect. If the distribution is skewed then it is likely that there is a an additional variable playing a role, for example aromaticity of bonds may skew the distribution of bonds corresponding to atom types within aromatic systems. Another example where the distribution might become skewed is a three-bonded N atom where there is an uncertainty as to its hybridization state; when nitrogen moves from *sp* ^2^ to *sp* ^3^ the corresponding bond lengths increase. These facts should be used to model such behaviour; this should be one of the subjects of future investigation.

**Figure 3:**
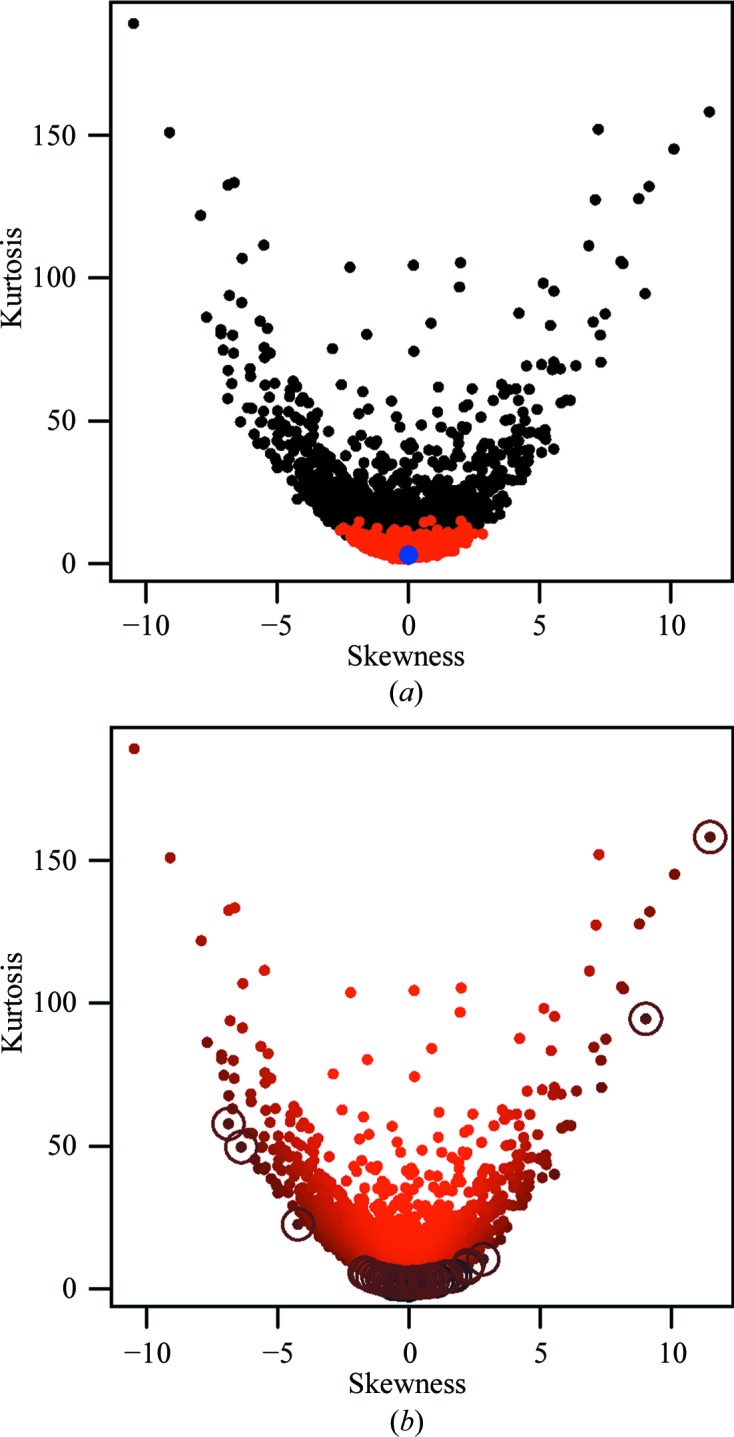
Plots showing kurtosis *versus* skewness for the 2996 bond classes in the *AceDRG* tables from June 2014. (*a*) shows data corresponding to all classes (black), as well as identifying classes that have no extreme outliers (red). Note that extreme outliers (*Z*-score > 5) are now removed by *AceDRG*. It is evident that classes with extreme outliers had a tendency to have a high absolute skewness and/or high kurtosis. For reference, a point corresponding to the normal distribution is also shown (blue). (*b*) shows the same plot, with observations coloured according to the multimodality index (which is a modified version of Sarle’s coefficient) using a colour gradient from black (high) to red (low). Classes with a high multimodality index have a particularly low kurtosis given the skewness. To illustrate this, points corresponding to the 50 bond classes with the highest multimodality indices are highlighted with circles. This figure was produced by *R* (R Core Team, 2013 ▸).

The skewness and kurtosis values were used to characterize the shapes of the bond-length distributions. Fig. 3 ▸ shows a skewness *versus* kurtosis plot.

We also used these values to calculate Sarle’s coefficient, which is a useful tool that can indicate bi/multimodality of distributions. The formulae for skewness (γ), kurtosis (*k*) and Sarle’s coefficient (*s*) are
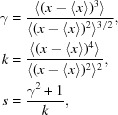
where *x* denotes a random variable and 〈.〉 denotes the sample average. In general, skewness is a measure of the departure of the distribution from symmetric distributions, and kurtosis is the departure of the distribution from normal. For symmetric distributions the skewness is 0. Kurtosis mainly gives an indication about the behaviour of the tails of the distributions. For the normal distribution, kurtosis is equal to three; for heavier-tailed distributions this value is larger and for rapidly falling distributions it is smaller. The main purpose of Sarle’s coefficient is to indicate bi/multimodality of the distributions, which is important in classifying atom types, bonds and angles. In our tests, we came to conclusion that using a modified version of Sarle’s coefficient, namely *s* = (γ^2^ + 2)/(2*k*), gives a better ranking of the distributions by multimodality. Specifically, we found that the standard version of Sarle’s coefficient had a tendency to bias towards identifying distributions with a low skewness; multimodal distributions with a higher skewness were never amongst the ranked hits. In contrast, the modified version of Sarle’s coefficient better identified distributions with a low kurtosis given the skewness, and was able to better identify multimodal distributions with both low and high skewness.

**Figure 4:**
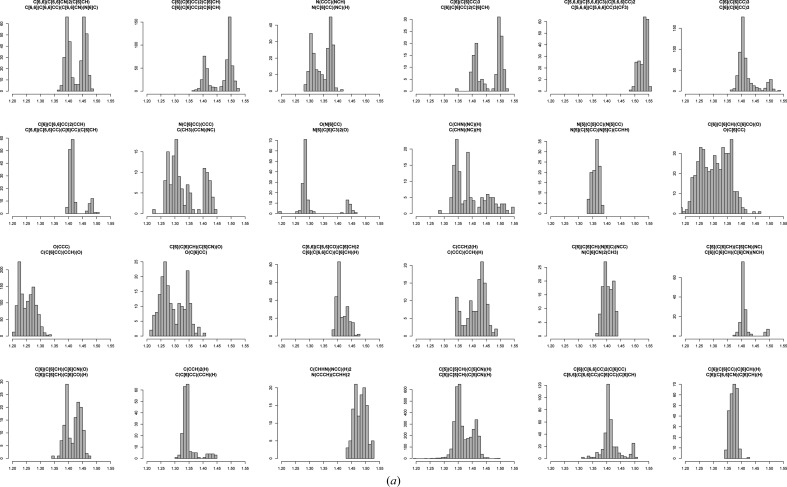

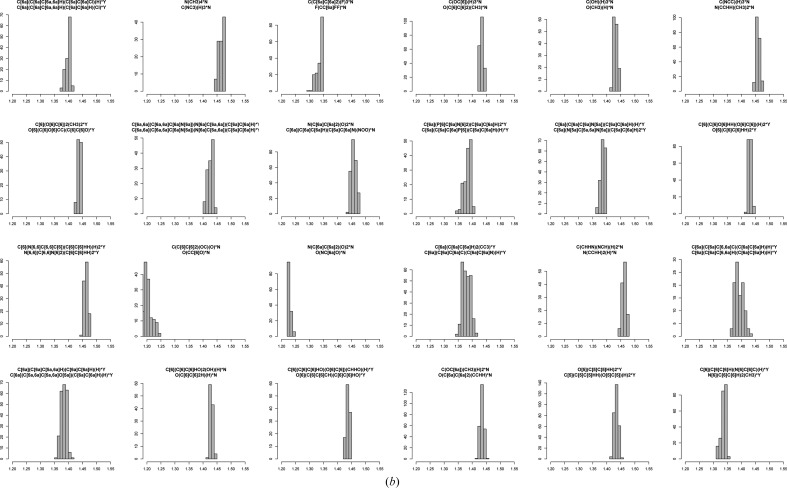
Histograms displaying bond-length distributions for the 24 bond classes with at least 100 observations that have the highest multimodality indices (a modified version of Sarle’s coefficient). The title of each histogram identifies the atom pair corresponding to the bond class. All histograms are shown on the same scale (1.2–1.55 Å). (*a*) corresponds to *AceDRG*-derived data generated in June 2014 and (*b*) to data generated in January 2015. In the earlier table, it is evident that there were a number of classes that clearly exhibited multimodal behaviour and comprised bond-length observations spanning a wide range of values. This indicates the original atom typing to be insufficient to describe the local chemical environments in a way suitable for purpose. In the latter table, even the most extremely multimodal classes span a comparatively shorter range of values, inevitably resulting in smaller derived standard deviations, and thus more accurate restraints. The class table from June 2014 comprised 268 882 bond classes, of which 2996 had at least 100 observations. In contrast, the class table from January 2015 comprised 169 362 bond classes, of which 1222 had at least 100 observations. This illustrates how the selection criteria have become more stringent from one table generation to the next. This figure was produced by *R* (R Core Team, 2013 ▸).

Fig. 4 ▸(*a*) shows the 24 bond classes with the highest modified Sarle’s coefficient. It should be noted that for our analysis we only considered bond classes with over 100 observations. As more entries are added these analyses will, in due course, be repeated.

## 4. Fine-tuning of atom types   

The statistical methods described in the previous section were applied for the *AceDRG*-derived bond observation and bond class tables. In addition to cleaning up the bond classes, the results were fed back to fine-tune the atom-type classes. There were two types of feedback.

(i) Selection of bonds and angles used for bond table classes were modified, *i.e.* stricter criteria were used by *AceDRG* to select the bonds to be used for mean and standard deviation calculations.
(ii) Findings were used to fine-tune atom classes and bond classes.

**Figure 5:**
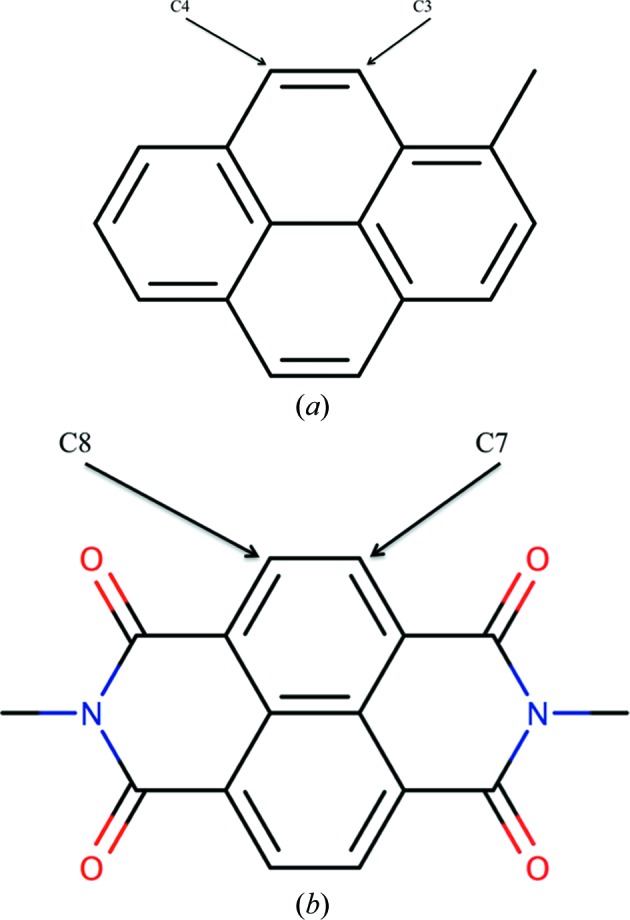
Fine-tuning of atom types. Chemical diagrams corresponding to portions of structures from the COD. (*a*) COD code 1502982. The distance between atoms C3 and C4 is 1.34 Å. (*b*) COD code 2223257. The distance between atoms C7 and C8 is 1.41 Å. In the early atom-type classification scheme both atom-type pairs belonged to the same bond class. After adding the third-neighbour information they belong to two different classes. The current atom types for these atoms are (*i.e.* the third neighbour information is added) C[6a](C[6a,6a]C[3x6a]C[6a])(C[6a]C[6a,6a]H)(H){1|N<3>,1|O<1>,3|C<3>} and C[6a](C[6a,6a]C[3x6a]C[6a])(C[6a]C[6a,6a]H)(H){1|H<1>,4|C<3>}. This figure was produced by *Marvin Sketch* (http://www.chemaxon.com).

This procedure was repeated four times, and the procedure will be repeated again in the future. Fig. 4 ▸ shows results corresponding to the bond tables after two iterations. Fig. 4 ▸(*a*) shows the worst 24 bond classes in the first table generated by *AceDRG*. Detailed analysis showed that atom types and bond classes should be modified further to remove some of the multimodality of the distributions. For example, it became clear that for the same type of atom pairs, whether they are within or between rings makes a difference to the bond-length distribution. Therefore, an additional descriptor was added to the bond classes to distinguish these cases. Further detailed analysis showed that the nature of the third-neighbour atoms affects the bond length (Fig. 5 ▸). This makes sense, as in the case of aromatic rings if the third neighbour is a double-bonded O atom then it is likely that one of the π electrons will be localized mostly for this bond, making neighbouring bonds more single-bonded in nature, and this will have an affect on the classified atoms as well as on the resulting average bond lengths.

## 5. Validation of ligand descriptions and feedback to *AceDRG*   

Further validation was performed on the *AceDRG* output dictionaries. They underwent wholesale testing in three ways. The first was to check that the atom types obeyed the internal rules. An independent *RDKit*-based program was written using the *Coot* libraries to generate atom types and compare them with the canonical *AceDRG* implementation. Where differences were found, the *AceDRG* atom typing was updated so that full consistency was achieved for the 3000+ non-hydrogen extended organic set atom types.

Secondly, *AceDRG* in dictionary-generator mode was run with several hundred monomers from the *REFMAC* monomer library as input. Alternative dictionary-generating tools (Smart *et al.*, 2011 ▸) and *pyrogen* (based on the *Coot* libraries) were run to generate corresponding dictionaries. The dictionaries were then compared using a bespoke tool and the major bond-length and angle target value outliers were analysed. This identified several edge cases (particularly in generalization) where the output of *AceDRG* was non-optimal. These issues were corrected and the comparisons were made again. Interestingly, there were several examples of large bond-length differences created by different ‘generalization’ methods and it was found that the reason was very ‘unusual’ chemistry, stemming from erroneous entries in the Chemical Component Dictionary (Feng *et al.*, 2004 ▸; Dimitropoulos *et al.*, 2006 ▸). These were reported back to the PDBe (Velankar *et al.*, 2016 ▸) and the entries were updated there.

Thirdly, the *AceDRG* dictionaries were used with *REFMAC* (Murshudov *et al.*, 2011 ▸) for refinement. Incorrectly specified chiral centres would not necessarily be highlighted by the previous test, but refinement with *REFMAC* would distort or change the ligand geometry from the deposited structure. Thus, deviations in atom position and deviations of density correlations and pre- and post-refinement comparisons *versus* *Mogul* highlighted further problems. These have subsequently been addressed.

## 6. Conclusions and future perspectives   

Repositories of experimentally derived crystal structures such as the COD and the CSD are valuable sources, allowing the general structural properties of small compounds, including molecular-geometry information, to be studied and organized. However, since researchers of varying experience, with the help of software, derive these structures, it can be expected that these sources contain some erroneous or unreliable data. Moreover, the purpose of small-molecule research in general is very different from that of structural biology, and thus we can expect that many ligands of interest in structural biology are ill-represented in these databases. For example, these databases contain many organometallic compounds with varying complexity. Only 7% of compounds from the PDB’s Chemical Component Dictionary contain metals. The selection of relevant crystal structures from these repositories is a challenging problem that this contribution attempts to deal with.

*AceDRG* and several auxiliary programs were designed and used together with the statistical package *R* (R Core Team, 2013 ▸) for the selection and validation of entries from the COD, as well as for the validation of derived data such as atom types, bond and angle classes. The results of statistical analyses were fed back to aid *AceDRG* atom-type and bond-class redefinition. The procedure of deriving atom types and bond classes and statistical analysis was performed in four iterations. This resulted in sufficiently fine-graded atom typing that is applicable to a wide range of ligands. We found that using indicators based on higher-order moments such as skewness, kurtosis and Sarle’s coefficient were valuable in the identification of unusual distributions of bond lengths within bond classes. Sarle’s coefficient in particular is a good indicator showing multimodality of the distribution. It is clear that if the distribution is multimodal then either the atom typing needs to be redefined or the wrong structures have been used.

Derived atom types, bond and angle classes, together with the corresponding tables, are now of sufficient quality and are used by *AceDRG* (Long *et al.*, 2017 ▸) to derive molecular-geometry information for a given ligand from basic molecular-geometry descriptions.

The results of *AceDRG* have also been validated against various sources of molecular-geometry information, including those derived using tools from *Mogul* (Bruno *et al.*, 2004 ▸). The results show that, in general, *AceDRG* produced descriptions that are in close agreement with those derived using *Mogul*, as can be expected given that both of them are based on small-molecule structural databases, albeit using different algorithms (Steiner & Tucker, 2017 ▸).

The current approaches to atom typing and validation have not been applied for metals. They pose special problems and require different types of analysis. Zheng *et al.* (2017 ▸) use an approach to deal with metal-containing compounds. This method is a variation on bond-valence theory (Brown, 2009 ▸), which seems to be a good approach for classification and the use of molecular-geometry information around metals. Even for this case it is necessary to validate crystal structures and molecules containing metals.

One of the often-ignored properties of any experimentally derived data is redundancy: often the same structure, or very similar ones, are studied by one or more research groups under different conditions to answer the same (or different) questions. This is a valuable technique for studies of the behaviour and properties of compounds. However, when properties that are common to all ligands are studied then such redundancy in the data does inevitably result in bias in the derived data. Future analysis will require redundancy analysis of the COD (and perhaps, in due course, the CSD). If such analysis is used then the data can be organized and analysed in a hierarchical manner: within the same class of compounds and between different classes of compounds. To perform such analysis, the derived molecules can be used and then, using subgraph isomorphism techniques, compounds can be classified or clustered. Each cluster (or class) of compounds can then be analysed in order to understand within-class properties (*e.g.* stereochemistry, conformations, bonds and angles) and between-class properties that can be applied for substructures (such as atom types defined by *AceDRG*) that are common among different classes.

We would like also to mention that in this work we do not consider the libration effect that causes bond-length shortening (Allen *et al.*, 1987 ▸), accounting for which may increase the accuracy of derived bond lengths and angles.

In general, we applaud that such rich databases as the COD are to be publicly available: it increases their value considerably by allowing a wide range of researchers to analyse them and to be able to make the derived data publicly available.

## Figures and Tables

**Table 1:** The numbers of atom-type, bond and angle classes

Atom classes	261057
Bond classes	561440
Observed bonds	5178340
Angle classes	1352226
Observed angles	8537384

**(a):** Crystal structure selection (as of September 2016). The number of surviving crystals for further use is around 46% of the total crystals in the COD.

Selection procedure	No. of excluded crystals	No. of surviving crystals
Total	0	366504
Remove powder diffraction	4184	362320
No info about maximum resolution	4896	357424
Resolution (*d*_max_ > 0.84)	38129	319295
*R* factor (>0.1)	134322	184973
Metals as a second neighbour of atoms, atoms collide (distance < 0.1 Å), half of the atoms have low occupancy (<1.0) *etc*.	17484	167489

**(b):** Molecule selection.

Selection procedure	No. of excluded molecules	No. of surviving molecules
Total no. of molecules	0	325764
At least one atom has low occupancy (<1.0)	59394	266370
Large variation of bonds between the same atom-type pairs	29926	236444
Different atom-type pairs have the same distance	6921	229523
Inconsistency between valence and the number of connections	4662	224861

**Table 3:** Default ‘bond radii’ of the atoms from the ‘extended organic set’ These are consensus values taken from Cordero *et al.* (2008 ▸), Pyykkö & Atsumi (2009*a* ▸,*b* ▸) and from the website http://periodictable.com/Properties/A/CovalentRadius.v.log.html. The values from these sources were adjusted to make sure that the molecular geometries of all validated structures from the COD could be reproduced.

Bonds	C	N	O	S	P	B	Se	H
Covalent radius (Å)	0.76	0.70	0.68	1.02	1.05	0.83	1.22	0.32

**Table 4:** Maximum valence of the ‘extended organic set’ of atoms (maximum number of connections allowed)

Element	H or halogens	B	C	N	O	S	P	Se	Si
Valence	1	4	4	4	2	4	4	4	4
